# A Lumped-Parameter Subject-Specific Model of Blood Volume Response to Fluid Infusion

**DOI:** 10.3389/fphys.2016.00390

**Published:** 2016-08-31

**Authors:** Ramin Bighamian, Andrew T. Reisner, Jin-Oh Hahn

**Affiliations:** ^1^Department of Mechanical Engineering, University of MarylandCollege Park, MD, USA; ^2^Department of Emergency Medicine, Massachusetts General HospitalBoston, MA, USA

**Keywords:** fluid shifts, blood volume, fluid resuscitation, mathematical model, lumped-parameter model, subject-specific model

## Abstract

This paper presents a lumped-parameter model that can reproduce blood volume response to fluid infusion. The model represents the fluid shift between the intravascular and interstitial compartments as the output of a hypothetical feedback controller that regulates the ratio between the volume changes in the intravascular and interstitial fluid at a target value (called “target volume ratio”). The model is characterized by only three parameters: the target volume ratio, feedback gain (specifying the speed of fluid shift), and initial blood volume. This model can obviate the need to incorporate complex mechanisms involved in the fluid shift in reproducing blood volume response to fluid infusion. The ability of the model to reproduce real-world blood volume response to fluid infusion was evaluated by fitting it to a series of data reported in the literature. The model reproduced the data accurately with average error and root-mean-squared error (RMSE) of 0.6 and 9.5% across crystalloid and colloid fluids when normalized by the underlying responses. Further, the parameters derived for the model showed physiologically plausible behaviors. It was concluded that this simple model may accurately reproduce a variety of blood volume responses to fluid infusion throughout different physiological states by fitting three parameters to a given dataset. This offers a tool that can quantify the fluid shift in a dataset given the measured fractional blood volumes.

## Introduction

The fluid shift between the intravascular and interstitial compartments is an essential mechanism of homeostasis, and a key determinant of the physiological response to circulatory pathology and medical therapy. The net fluid shift is determined by the summary action occurring across the body's massive network of microvasculature, and the determinants of flow for each microscopic segment are complex, including the permeability of the vessels and the local Starling forces (i.e., hydrostatic and oncotic pressure gradients). Each of these determinants can be, in turn, altered by a wide range of other factors, including the vasomotion of upstream and downstream vessels, lymphatic flow, and a myriad of endocrine and exocrine signals that affect the preceding determinants.

Given the undeniable complexity that underlies fluid shift between the intravascular and interstitial compartments, it can be challenging to mathematically model the fluid shift in an individual subject or patient. Existing models of fluid shift can reproduce the volume changes in the blood and interstitial fluid (Cervera and Moss, 1974; Pirkle and Gann, 1975; Hedlund et al., 1988; Mazzoni et al., 1988; Arturson et al., 1989; Carlson et al., 1996; Gyenge et al., 2003; Tatara et al., 2007) and even intracellular fluid (Hedlund et al., 1988; Mazzoni et al., 1988; Arturson et al., 1989; Carlson et al., 1996; Ursino and Innocenti, 1997; Gyenge et al., 2003; Fernandez de Canete and Del Saz Huang, 2010; Siam et al., 2013). However, there are so many disparate factors that, without exhaustive and impractical measurements, it is not possible to comprehensively characterize the fluid shift associated with an individual. A reasonable alternative is to use “typical” values for certain parameters (i.e., values that represent average values in a population) (Champion et al., 1975; Lewis, 1986; Wears and Winton, 1990; Mardel et al., 1995; Simpson et al., 1996; Hirshberg et al., 2006; Saito et al., 2013) but this is no longer an individualized model. Recently reported volume kinetic models (Ståhle et al., 1997; Svensén and Hahn, 1997; Drobin and Hahn, 1999, 2002; Hahn, 2010) may offer value via a tradeoff between simplicity and transparency. However, these models still have weakness in terms of physiological transparency. As an example, volume kinetic models dictate that the ratio between the changes in the intravascular and interstitial volumes depends only on their baseline values (see Appendix for details), which is an oversimplification of the known physiology that the ratio actually varies with the level of intravascular and interstitial volumes as well as physiological conditions (Guyton et al., 1975). To overcome the challenges in modeling the fluid shift in an individual, we sought to develop a lumped-parameter model that could reproduce the fluid shift at the macroscopic level without microscopic details between the intravascular and interstitial compartments. Specifically, we sought a model which is simple enough to be fitted to individual subjects with only a rudimentary set of measurements, and at the same time accurate enough to characterize that subject's response to fluid infusion. The ability of the model to reproduce real-world blood volume response to fluid infusion was evaluated by fitting it to a series of data.

## Materials and methods

### Development of a lumped-parameter model of blood volume response to fluid infusion

The proposed lumped-parameter model involves only three parameters to describe an individual's fluid shift after fluid infusion: target volume ratio (i.e., ratio between the volume changes in the intravascular and interstitial fluids), α; feedback gain (specifying the speed of fluid shift), K; and initial blood volume, V_B0_.

The first parameter of the proposed model is α, which varies depending on the overall physiological state of the subject. By way of background, the volume of fluid stored in the intravascular, interstitial and intracellular compartments is determined by the vessel permeability and the hydraulic and osmotic pressure gradients at the capillary walls and cell membranes. From microscopic standpoint, the kinetics of a number of ions and proteins as well as the pressure-volume relationships of the fluid compartments determine the hydraulic and osmotic pressure gradients, and therefore, the fluid volume stored in each compartment. However, from macroscopic standpoint, the consequence of the interaction among these complex mechanisms is that the ratio between the volume changes in the intravascular and interstitial fluid is summarized by a constant parameter value, denoted here as α (Guyton et al., 1975). Typically, interstitial fluid volume changes 2–3 times as much as intravascular volume changes (i.e., α = 2–3) when total blood volume is increased, up to a critical blood volume level. Beyond this level, however, majority of the fluid infused to the body is not stored in the intravascular compartment but is shifted to the interstitial compartment (i.e., α≫2–3) (Guyton et al., 1975; Bajwa and Kulshrestha, 2012). In sum, the fluid shift between intravascular and interstitial compartments, which is due to an array of complex physiological processes, may be viewed as the output of a hypothetical feedback controller that regulates the volume changes in the intravascular and interstitial fluid at a target ratio 1:α.

**Figure 1:**
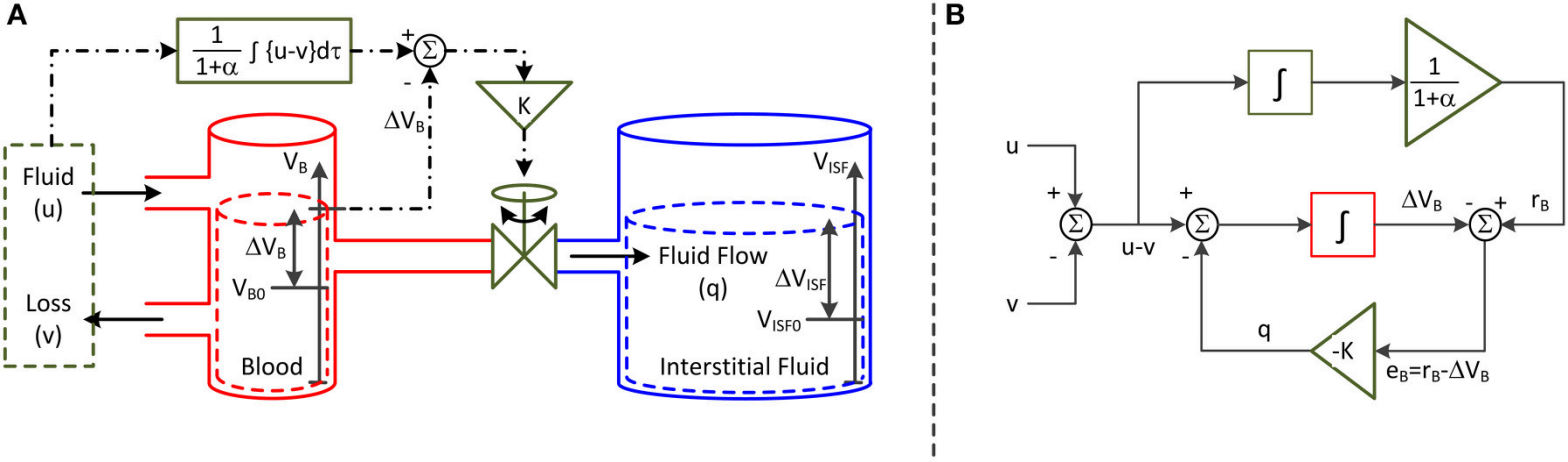
**Lumped-parameter model of blood volume response to fluid infusion**. **(A)** Feedback control system analogy via connected compartments. The left and right compartments represent the intravascular and interstitial compartments, while the valve represents a summary of all the physiologic processes that produce the fluid shift between the two compartments. The fluid infusion (u) and loss (v; e.g., hemorrhage and urine) act on the intravascular compartment to alter the blood volume (V_B_), which in turn alters the interstitial fluid volume (V_ISF_). The magnitude of valve opening is a function of the discrepancy between the target versus actual changes in blood volume. **(B)** Mathematical model. The fluid infusion (u) and loss (v; e.g., hemorrhage and urine) are the inputs to the model, while the change in blood volume (ΔV_B_) is the output. The objective of the feedback controller is to retain the 1/(1+α) fraction of the inputted fluid volume in the intravascular compartment while shifting the remaining α/(1 + α) fraction to the interstitial compartment in the steady state. The fluid shift from the intravascular to interstitial compartment (q) acts as feedback control to steer ΔV_B_ to the target change in blood volume (r_B_).

The second parameter of the model is K, which specifies the speed of fluid shift, i.e., the rate of fluid shift between the intravascular and interstitial compartments. K also varies depending on the physiological state of the subject. This parameter is predicated on a feedback control system analogy shown in Figure 1A. The left and right compartments represent the intravascular and interstitial compartments, respectively, while the valve represents a summary of all the physiological processes that produce the fluid shift between the two compartments. In this analogy, it is both fluid infusion (u) and loss (v; e.g., hemorrhage and urine) that act on the intravascular compartment to alter blood volume (V_B_). The magnitude of the valve opening is a function of the discrepancy between the target versus actual changes in V_B_: there is an increased rate of fluid shift,q, between the intravascular and interstitial compartments when the discrepancy between target versus actual V_B_ grows larger.

The third parameter of the model is V_B0_. Its role is to normalize V_B_ to yield its fractional change from the initial V_B_ ((V_B_ − V_B0_)/V_B0_) as the output of the model. Considering that fractional change in V_B_, rather than its absolute change, is reported in many experimental protocols (Ståhle et al., 1997; Svensén and Hahn, 1997; Drobin and Hahn, 1999, 2002; Hedin and Hahn, 2005; Hahn, 2010; Hahn et al., 2013), the above normalization allows the model to readily analyze experimental data obtained from such protocols.

The representation in Figure 1A can be formalized into the mathematical model (or block diagram) shown in Figure 1B. Both u and v are the inputs to the model, while the change in V_B_ from V_B0_ (ΔV_B_ = V_B_ − V_B0_) is the output. The objective of the feedback controller is to retain the 1/(1+α) fraction of the inputted fluid volume in the intravascular compartment while shifting the remaining α/(1+α) fraction to the interstitial compartment in the steady state. q acts as feedback control to steer ΔV_B_ to the target change in V_B_ (r_B_). In this way, the steady-state volume changes associated with the intravascular and interstitial fluid achieve the 1:α ratio.

The governing equation associated with this mathematical model is derived as follows. The rate of change in V_B_ is given by the following ordinary differential equation:

(1) $$\Delta {\dot{\text{V}}}_{\text{B}}\left(\text{t}\right)=\text{u}\left(\text{t}\right)-\text{v}\left(\text{t}\right)-\text{q}\left(\text{t}\right)$$

q is formulated to eliminate the discrepancy between the target versus actual changes in V_B_. As described above, r_B_ in response to u and v is the 1/(1 + α) fraction of the accumulated resultant fluid intake u − v:

(2) $${\text{r}}_{\text{B}}\left(\text{t}\right)=\frac{1}{1+\alpha }\int_{0}^{\text{t}}\left[\text{u}\left(\tau \right)-\text{v}\left(\tau \right)\right]\text{d}\tau$$

To capture the macroscopic behavior of q with a possibly simplest mathematical expression, q is modeled to be proportional to e_B_ = r_B_ − ΔV_B_:

(3) $$\text{q}\left(\text{t}\right)=-{\text{Ke}}_{\text{B}}\left(\text{t}\right)$$

This expression physically means that (1) q shifts from the intravascular to interstitial compartment if r_B_ < ΔV_B_ while from interstitial to intravascular compartment if r_B_ > ΔV_B_, and that (2) q increases as the discrepancy between r_B_ and ΔV_B_ increases. Combining (Equations 1–3) yields the following ordinary differential equation as the lumped-parameter model that relates u − v to ΔV_B_:

(4) $$\Delta {\overset{¨}{\text{V}}}_{\text{B}}\left(\text{t}\right)+\text{K}\Delta {\dot{\text{V}}}_{\text{B}}\left(\text{t}\right)=\left[\dot{\text{u}}\left(\text{t}\right)-\dot{\text{v}}\left(\text{t}\right)\right]+\frac{\text{K}\left[\text{u}\left(\text{t}\right)-\text{v}\left(\text{t}\right)\right]}{\left(\text{1}+\alpha \right)}$$

where $\frac{\text{d}}{\text{dt}}\left[\text{u}\left(\text{t}\right)-\text{v}\left(\text{t}\right)\right]=\overset{\cdot }{\text{u}}\left(\text{t}\right)-\overset{\cdot }{\text{v}}\left(\text{t}\right)$ was used. To rewrite (Equation 4) in terms of the fractional V_B_ response, ΔV_B_ must be normalized by V_B0_. Dividing both sides of (Equation 4) by V_B0_ yields (where ${\overset{⌣}{\text{V}}}_{\text{B}}(\text{t})=\Delta {\text{V}}_{\text{B}}\left(\text{t}\right)/{\text{V}}_{\text{B0}}$):

(5) $${\overset{\overset{¨}{⌣}}{\text{V}}}_{\text{B}}\left(\text{t}\right)+\text{K}{\overset{\dot{⌣}}{\text{V}}}_{\text{B}}\left(\text{t}\right)=\frac{\left[\dot{\text{u}}\left(\text{t}\right)-\dot{\text{v}}\left(\text{t}\right)\right]}{{\text{V}}_{\text{B0}}}+\frac{\text{K}\left[\text{u}\left(\text{t}\right)-\text{v}\left(\text{t}\right)\right]}{{\text{V}}_{\text{B0}}\left(\text{1}+\alpha \right)}$$

The parameters in the model (Equation 5) can be uniquely determined if the data are informative (Ljung, 1999). To illustrate, (Equation 5) can be transformed to the following linear regression:

(6) $${\ddot{\overset{⌣}{\text{V}}}}_{\text{B}}(\text{t})=\left[\begin{array}{ccc} \text{K} & \frac{\text{1}}{{\text{V}}_{\text{B}0}} & \frac{\text{K}}{{\text{V}}_{\text{B}0}(\text{1}+\alpha )} \end{array}\right]\;\left[\begin{array}{c} {\dot{\overset{⌣}{\text{V}}}}_{\text{B}}(\text{t})\\ \dot{\text{u}}(\text{t})-\dot{\text{v}}(\text{t})\\ \text{u}(\text{t})-\text{v}(\text{t}) \end{array}\right]=\theta^{\text{T}}\phi (\text{t})$$

The vector θ in a linear regression is identifiable (Ljung, 1999) (meaning its elements can be uniquely determined provided the data are informative). Hence, all the parameters can be uniquely determined from θ once it is determined via, e.g., the standard least-squares technique (Ljung, 1999) or even a numerical optimization technique (Storn and Price, 1997): K from the first element of θ; V_B0_ from the second element of θ; and α from the third element of θ using K and V_B0_ thus determined.

### Model evaluation method

The lumped-parameter model detailed above is intended to summarize V_B_ response to u and v in an individual subject or patient using just three parameters specific to the individual. However, it is possible that the underlying physiology is too complex to be adequately reproduced by such a simple model. Hence, we sought to evaluate the ability of this lumped-parameter model to reproduce real-world blood volume response to fluid infusion by fitting it to a multitude of different experimental datasets, to assess whether or not such a simple model is capable of accurately reproducing the experimental data.

#### Experimental data

To analyze the model, a series of datasets reported in the literature were used that had ${\overset{⌣}{\text{V}}}_{\text{B}}$ response (versus time) to u and v. In all the datasets used in this study, ${\overset{⌣}{\text{V}}}_{\text{B}}$ was measured using hemoglobin as the tracer substance. Assuming that the mass of the tracer substance remains constant, an increase in V_B_ results in a decrease in the concentration of the tracer substance in the blood. Hence, ${\overset{⌣}{\text{V}}}_{\text{B}}$ can be computed from the change in the blood hemoglobin concentration as follows (Drobin and Hahn, 1999, 2002; Hahn, 2010):

(7) $$\begin{array}{l} {\overset{⌣}{\text{V}}}_{\text{B}}\left(\text{t}\right)=\frac{\Delta {\text{V}}_{\text{B}}\left(\text{t}\right)}{{\text{V}}_{\text{B0}}}=\frac{{\text{V}}_{\text{B}}\left(\text{t}\right)-{\text{V}}_{\text{B0}}}{{\text{V}}_{\text{B0}}}\\ \;=\frac{1}{\text{1}-\text{Hct}\left(\text{t}\right)}\frac{\text{Hgb}\left(0\right)-\text{Hgb}\left(\text{t}\right)}{\text{Hgb}\left(\text{t}\right)} \end{array}$$

where Hct(t) is the hematocrit at time t, and Hgb(t) is hemoglobin concentration at time t. The datasets used in this study included (1) ${\overset{⌣}{\text{V}}}_{\text{B}}$ response to bolus infusion of fluid under different V_B_ states (Dataset 1 Drobin and Hahn, 1999); (2) ${\overset{⌣}{\text{V}}}_{\text{B}}$ response to bolus infusion of crystalloid fluids (Dataset 2 Drobin and Hahn, 2002); and (3) ${\overset{⌣}{\text{V}}}_{\text{B}}$ response to bolus infusion of colloid fluids (Dataset 3 Hedin and Hahn, 2005).

From the aforementioned reports (Drobin and Hahn, 1999, 2002; Hedin and Hahn, 2005), the volume of fluid infused and ${\overset{⌣}{\text{V}}}_{\text{B}}$ response were extracted at 10 min intervals as the average of the maximum and minimum responses across all the subjects (although the model is intended for use in individual subjects, average responses were considered here because the response associated with each subject could not be extracted from the visually presented data in these reports). The urine excretion rate was estimated by dividing the total urine volume by the study duration (infusion time + post-infusion observation time) based on the simplifying assumption that it remained constant, because only the total urine volumes were available in these reports.

**Figure 2:**
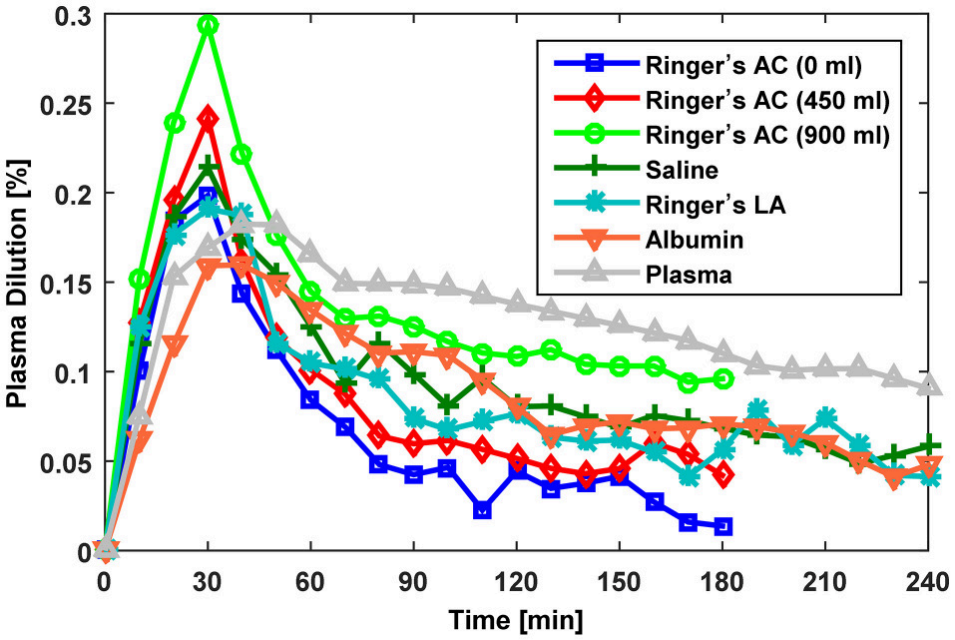
**Measured fractional blood volume responses to fluid used for evaluation of the lumped-parameter model**. Ringer's AC, ringer's acetate (Dataset 1; Drobin and Hahn, 1999); Saline,0.9% saline (Dataset 2; Drobin and Hahn, 2002); Ringer's LA, ringer's lactate (Dataset 2; Drobin and Hahn, 2002); Albumin, 5% albumin (Dataset 3; Hedin and Hahn, 2005); Autologous Plasma, autologous plasma (Dataset 3; Hedin and Hahn, 2005).

Overall, we studied a total of seven distinct protocols summarized in Table 1: in Dataset 1, there were three protocols (with 0, 450, and 900 ml pre-infusion hemorrhage); in Dataset 2, there were two protocols (with infusions of 0.9% saline and ringer's lactate); and in Dataset 3, there were two protocols (with infusions of 5% albumin and autologous plasma). The measured ${\overset{⌣}{\text{V}}}_{\text{B}}$ responses associated with all the datasets are shown in Figure 2.

**Table 1:** **Datasets used for model evaluation**.

	**Dataset 1 (Drobin and Hahn, 1999)**	**Dataset 2 (Drobin and Hahn, 2002)**	**Dataset 3 (Hedin and Hahn, 2005)**
Number of subjects	10 (Humans)	10 (Humans)	15 (Humans)
Age (min-max)	23–33 year	24–44 year	18–36 year
Weight (min-max)	65–85 kg	72–95 kg	70–94 kg
Fluid infused	Crystalloid (Ringer's Acetate)	Crystalloid (Saline and Ringer's Lactate)	Colloid (Albumin and Autologous Plasma)
Infused volume	25 ml/kg	25 ml/kg	10 ml/kg
Infusion time	30 min	30 min	30 min
Observation time (Post-infusion)	150 min	210 min	450 min
Hemorrhage volume (Pre-infusion)	0 ml/450 ml/900 ml	0 ml	0 ml

#### Model fitting

Fitting the model to each dataset meant estimating the values of the three parameters α, K and **V**_B0_ associated with each dataset. First, Equation (5) was discretized into a difference equation using the Euler's method, where T_S_ is sampling interval (Nise, 2010):

(8) $$\begin{array}{l} {\overset{⌣}{\text{V}}}_{\text{B}}\left(\text{i}\right)=2{\overset{⌣}{\text{V}}}_{\text{B}}\left(\text{i}-\text{1}\right)-{\overset{⌣}{\text{V}}}_{\text{B}}\left(\text{i}-\text{2}\right)\\ \;-\;{\text{KT}}_{\text{S}}\left[{\overset{⌣}{\text{V}}}_{\text{B}}\left(\text{i}-\text{1}\right)-{\overset{⌣}{\text{V}}}_{\text{B}}\left(\text{i}-\text{2}\right)\right]\\ \;+\;\frac{{\text{T}}_{\text{S}}}{{\text{V}}_{\text{B0}}}\left\{\left[\text{u}\left(\text{i}-\text{1}\right)-\text{u}\left(\text{i}-\text{2}\right)\right]-\left[\text{v}\left(\text{i}-\text{1}\right)-\text{v}\left(\text{i}-\text{2}\right)\right]\right\}\\ \;+\;\frac{{\text{KT}}_{\text{S}}^{2}}{{\text{V}}_{\text{B0}}\left(\text{1}+\alpha \right)}\left[\text{u}\left(\text{i}-\text{2}\right)-\text{v}\left(\text{i}-\text{2}\right)\right] \end{array}$$

Second, an optimization problem was formulated to derive the set of optimal parameters that minimize the error between ${\overset{⌣}{\text{V}}}_{\text{B}}$ and its model-reproduced counterpart ${\hat{\overset{⌣}{\text{V}}}}_{\text{B}}$:

(9) $$\Theta^{*}=\{\alpha^{*},{\text{K}}^{*},{\text{V}}_{\text{B}0}^{*}\}=\text{arg }\underset{\Theta }{\min}\;\sum_{\text{i}=1}^{\text{N}}{\left[{\overset{⌣}{\text{V}}}_{\text{B}}(\text{i})-{\hat{\overset{⌣}{\text{V}}}}_{\text{B}}(\text{i})\right]}^{2}$$

where Θ is a vector of (unknown) parameters, Θ^*^ is the optimal Θ, ${\hat{\overset{⌣}{\text{V}}}}_{\text{B}}(\text{i})$ is model-reproduced ${\overset{⌣}{\text{V}}}_{\text{B}}$(i) at a discrete time instant i (i = 1⋯N) for a protocol. For a given set of Θ, ${\hat{\overset{⌣}{\text{V}}}}_{\text{B}}$ was computed by inputting u and v data to (Equation 8). The optimization problem (Equation 9) was solved using MATLAB's Optimization Toolbox (MathWorks, MA, USA).

#### Model testing

First, the ability of the model to reproduce ${\hat{\overset{⌣}{\text{V}}}}_{\text{B}}(\text{i})$ data was assessed by analyzing the goodness of fit associated with each protocol, in terms of sample-by-sample error normalized by the average ${\overset{⌣}{\text{V}}}_{\text{B}}$ (e) and root-mean-squared error normalized by the average ${\overset{⌣}{\text{V}}}_{\text{B}}$ (RMSNE):

(10) $$\begin{array}{l} \text{ e}(\text{i})=\frac{{\overset{⌣}{\text{V}}}_{\text{B}}(\text{i})-{\hat{\overset{⌣}{\text{V}}}}_{\text{B}}(\text{i},\Theta^{*})}{\bar{{\overset{⌣}{\text{V}}}_{\text{B}}(\text{i})}},\text{ i}=1,\cdots ,\text{N}\\ \text{RMSNE}\;=\frac{1}{\bar{{\overset{⌣}{\text{V}}}_{\text{B}}(\text{i})}}\sqrt{\frac{\sum_{\text{i }=\;1}^{\text{N}}{\left[{\overset{⌣}{\text{V}}}_{\text{B}}(\text{i})-{\hat{\overset{⌣}{\text{V}}}}_{\text{B}}(\text{i},\Theta^{*})\right]}^{2}}{\text{N}}} \end{array}$$

where $\bar{{\overset{⌣}{\text{V}}}_{\text{B}}(\text{i})}$ is the average ${\overset{⌣}{\text{V}}}_{\text{B}}$ response over the study duration, and ${\hat{\overset{⌣}{\text{V}}}}_{\text{B}}(\text{i},\;\Theta^{*})$ is ${\overset{⌣}{\text{V}}}_{\text{B}}$ reproduced by inputting u and v data to (Equation 8) characterized by Θ^*^.

Second, the intravascular and interstitial fluid volume responses to u and v were qualitatively compared across different protocols. $\Delta {\hat{\text{V}}}_{\text{B}}\left(\text{i},\Theta^{*}\right)$ associated with each protocol was estimated by $\Delta {\hat{\text{V}}}_{\text{B}}(\text{i},\;\Theta^{*})={\text{V}}_{\text{B}0}^{*}\cdot {\hat{\overset{⌣}{\text{V}}}}_{\text{B}}(\text{i},\;\Theta^{*})$, while the change in interstitial fluid volume $\Delta {\hat{\text{V}}}_{\text{ISF}}\left(\text{i},\Theta^{*}\right)$ was estimated as the numerical integration of q(i, Θ^*^):

(11) $$\begin{array}{l} \Delta {\hat{\text{V}}}_{\text{ISF}}\left(\text{i},\Theta^{*}\right)=\sum_{\text{n = 1}}^{\text{i}}\text{q}\left(\text{n},\Theta^{*}\right)=\sum_{\text{n}=1}^{\text{i}}-{\text{K}}^{*}{\text{e}}_{\text{B}}\left(\text{n},\Theta^{*}\right)\\ \;=\sum_{\text{n}=1}^{\text{i}}-{\text{K}}^{*}\left[{\text{r}}_{\text{B}}\left(\text{n}\right)-\Delta {\hat{\text{V}}}_{\text{B}}\left(\text{n},\Theta^{*}\right)\right] \end{array}$$

Third, the following hypotheses on the parameters were generated to gauge if they were meaningfully derived by the model fitting: (1) α^*^ decreases as pre-infusion hemorrhage increases; (2) ${\text{V}}_{\text{B}0}^{*}$ decreases as pre-infusion hemorrhage increases; and (3) α^*^ is larger in crystalloids than in colloids. These hypotheses were tested by examining the values of α^*^ and ${\text{V}}_{\text{B}0}^{*}$.

### Parametric sensitivity analysis

The parametric sensitivity analysis was performed in the frequency domain. The sensitivity functions associated with the model (Equation 5) to V_B0_, K, and α were derived as the normalized partial derivatives of G(jω), the frequency response function associated with (Equation 5), with respect to V_B0_, K, and α:

(12) $$\begin{array}{l} {\text{S}}_{{\text{V}}_{\text{B0}}}\left(\text{j}\omega \right)=\frac{{\text{V}}_{\text{B}0}}{\text{G}\left(\text{j}\omega \right)}{\frac{\partial \text{G}\left(\text{j}\omega \right)}{\partial {\text{V}}_{\text{B}0}}|}_{\Theta \;=\;\Theta^{*}}=-\;1\\ {\text{S}}_{\text{K}}\left(\text{j}\omega \right)=\frac{\text{K}}{\text{G}\left(\text{j}\omega \right)}{\frac{\partial \text{G}\left(\text{j}\omega \right)}{\partial \text{K}}|}_{\Theta \;=\;\Theta^{*}}=\frac{-{\text{K}}^{*}\alpha^{*}{\left(\text{j}\omega \right)}^{2}}{\left(1+\alpha^{*}\right){\left(\text{j}\omega \right)}^{3}+\;{\text{K}}^{*}\left(2+\alpha^{*}\right){\left(\text{j}\omega \right)}^{2}+{\text{K}}^{*2}\left(\text{j}\omega \right)}\\ {\text{S}}_{\alpha }\left(\text{j}\omega \right)=\frac{\alpha }{\text{G}\left(\text{j}\omega \right)}{\frac{\partial \text{G}\left(\text{j}\omega \right)}{\partial \alpha }|}_{\Theta \;=\;\Theta^{*}}=\frac{-\alpha^{*}}{\left(1+\;\alpha^{*}\right)}\frac{{\text{K}}^{*}}{\left(1+\;\alpha^{*}\right)\left(\text{j}\omega \right)+{\text{K}}^{*}} \end{array}$$

Then, the frequency responses of these sensitivity functions were examined to elucidate how V_B0_, K, and α are tuned to fit the model to the data on ${\overset{⌣}{\text{V}}}_{\text{B}}$ response to u and v.

## Results

Table 2 summarizes the results of data analysis, including the estimated parameters for each dataset and the associated error metrics. Overall, the model reproduced the data accurately with average normalized error and RMSNE of 0.6 and 9.5% across crystalloid and colloid fluids.

**Table 2:** **Data analysis results: estimated parameters and error metrics**.

	**Infusion type**	${\text{K}}_{\text{P}}^{*}$	${\text{V}}_{\text{B}0}^{*}$[l]	α^*^	**Error[%]**	**RMSE[%]**
Dataset 1	Ringer's AC (000 ml)	0.052	4.86	4.45	0.49 ± 14.1	13.3
	Ringer's AC (450 ml)	0.065	3.88	4.00	0.57 ± 7.93	7.52
	Ringer's AC (900 ml)	0.076	3.27	2.57	0.38 ± 4.04	3.84
Dataset 2	Saline	0.047	5.56	2.35	0.46 ± 10.0	9.62
	Ringer's LA	0.069	5.03	2.39	1.81 ± 17.6	16.9
Dataset 3	Albumin	0.028	3.94	0.45	0.05 ± 9.09	8.73
	Autologous Plasma	0.147	3.11	0.34	0.62 ± 6.49	6.25

${\text{K}}_{P}^{*}$, $V_{\text{B0}}^{*}$, and α^*^ are the estimated K_P_, V_B0_, and α associated with each dataset, obtained by solving the optimization problem (Equation 8) for each dataset. Ringer's AC, ringer's acetate. Ringer's LA, ringer's lactate.

**Figure 3:**
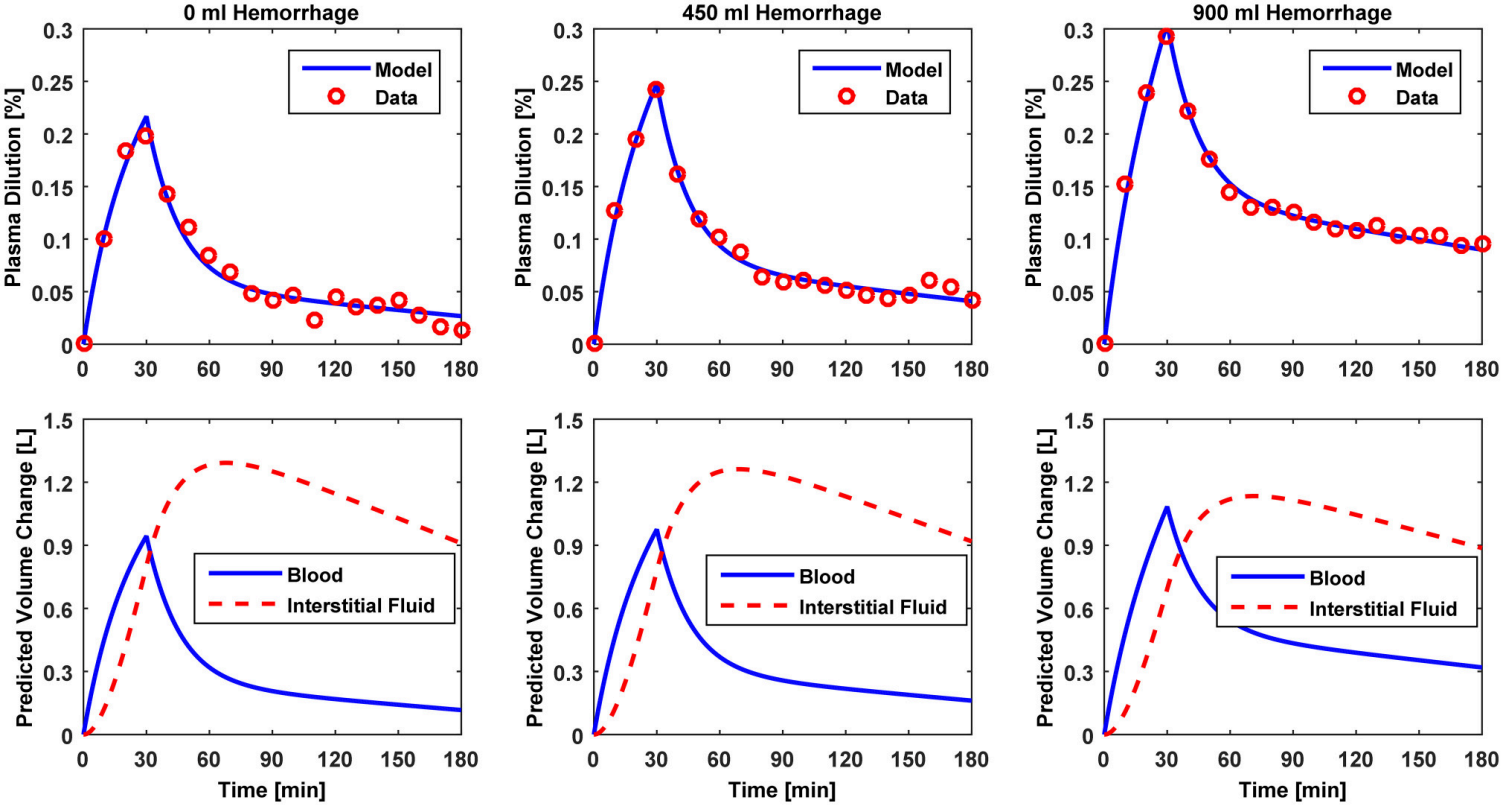
**Model evaluation for Dataset 1 (Drobin and Hahn, 1999)**. Upper panel, Measured fractional blood volume responses to ringer's acetate (“Data”) under different pre-infusion hemorrhage states, versus response reproduced by the lumped-parameter model (“Model”). Lower panel, Model-reproduced changes in blood and interstitial fluid volumes. 1st column, 0 ml pre-infusion hemorrhage; 2nd column, 450 ml pre-infusion hemorrhage; 3rd column, 900 ml pre-infusion hemorrhage.

**Figure 4:**
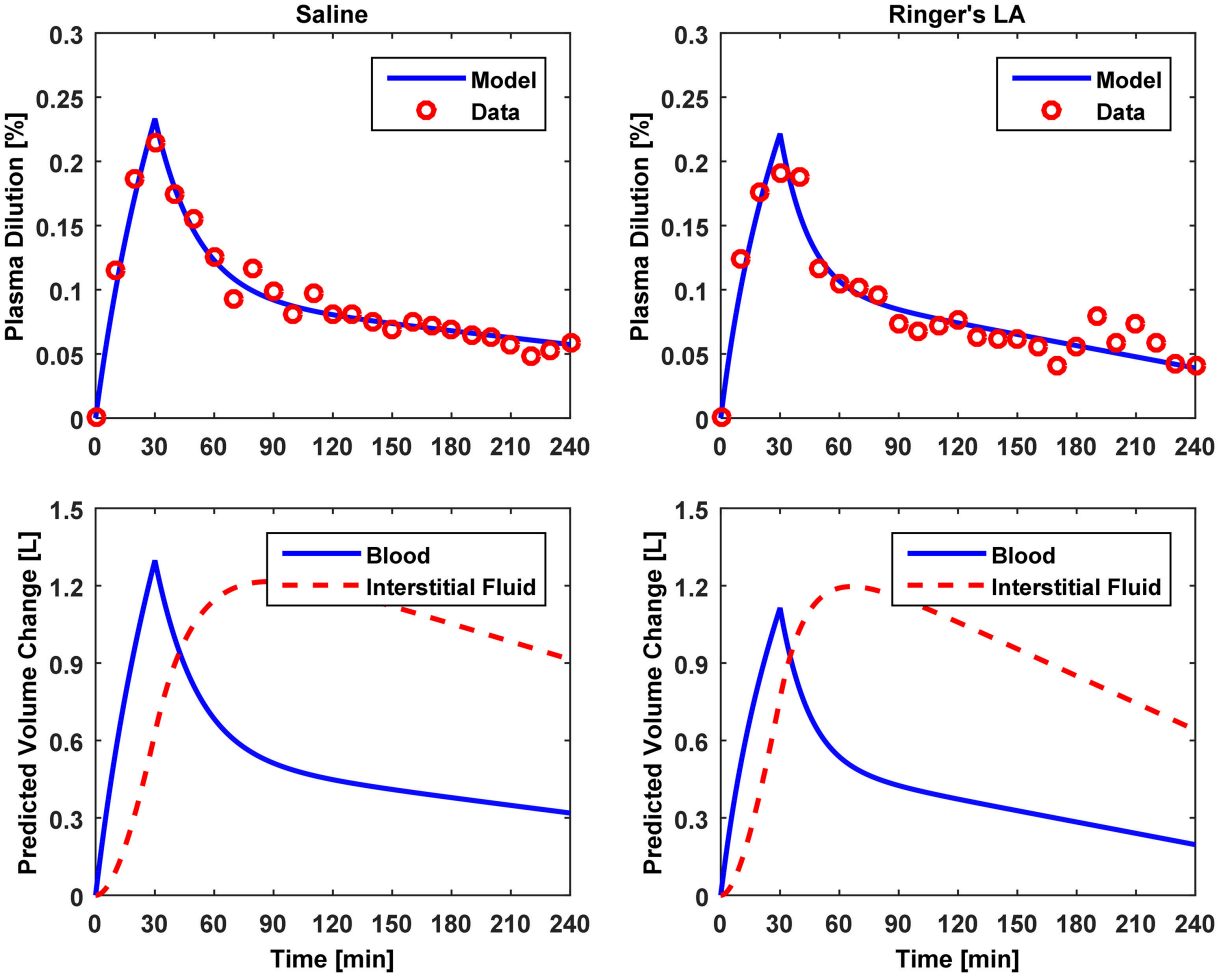
**Model evaluation for Dataset 2 (Drobin and Hahn, 2002)**. Upper panel, Measured fractional blood volume responses to crystalloid fluids (“Data”), versus response reproduced by the lumped-parameter model (“Model”). Lower panel, Model-reproduced changes in blood and interstitial fluid volumes. 1st column, 0.9% saline; 2nd column, ringer's lactate.

**Figure 5:**
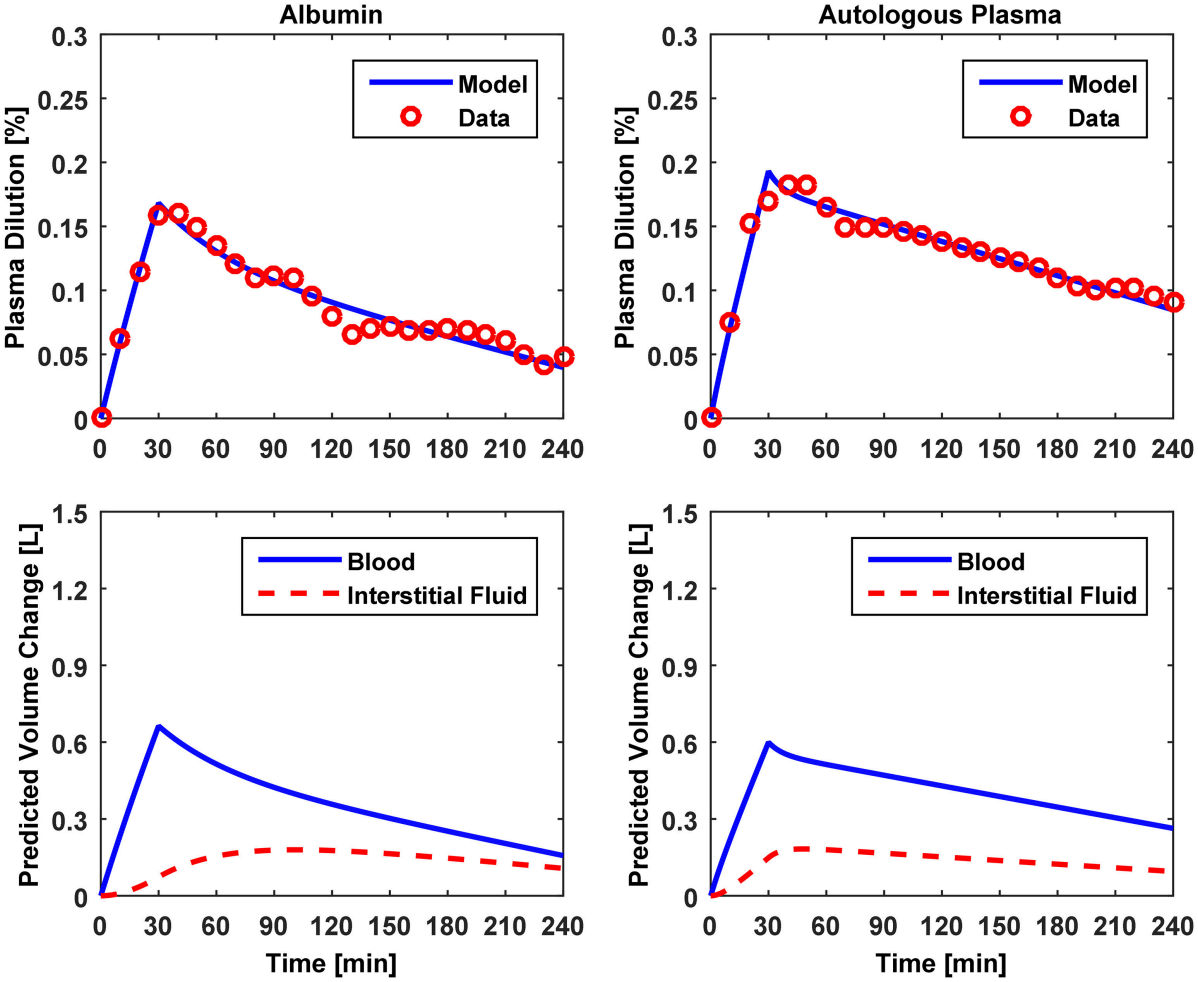
**Model evaluation for Dataset 3 (Hedin and Hahn, 2005)**. Upper panel, Measured fractional blood volume responses to colloid fluids (“Data”), versus response reproduced by the lumped-parameter model (“Model”). Lower panel, Model-reproduced changes in blood and interstitial fluid volumes. 1st column, 5% albumin; 2nd column, autologous plasma.

Figures 3–5 show ${\overset{⌣}{\text{V}}}_{\text{B}}$ responses reproduced by the model, together with the measured data, associated with Datasets 1, 2, and 3, respectively. The upper and lower panels present the measured versus model-reproduced ${\overset{⌣}{\text{V}}}_{\text{B}}$ responses as well as $\Delta {\hat{\text{V}}}_{\text{B}}$ and $\Delta {\hat{\text{V}}}_{\text{ISF}}$.

**Figure 6:**
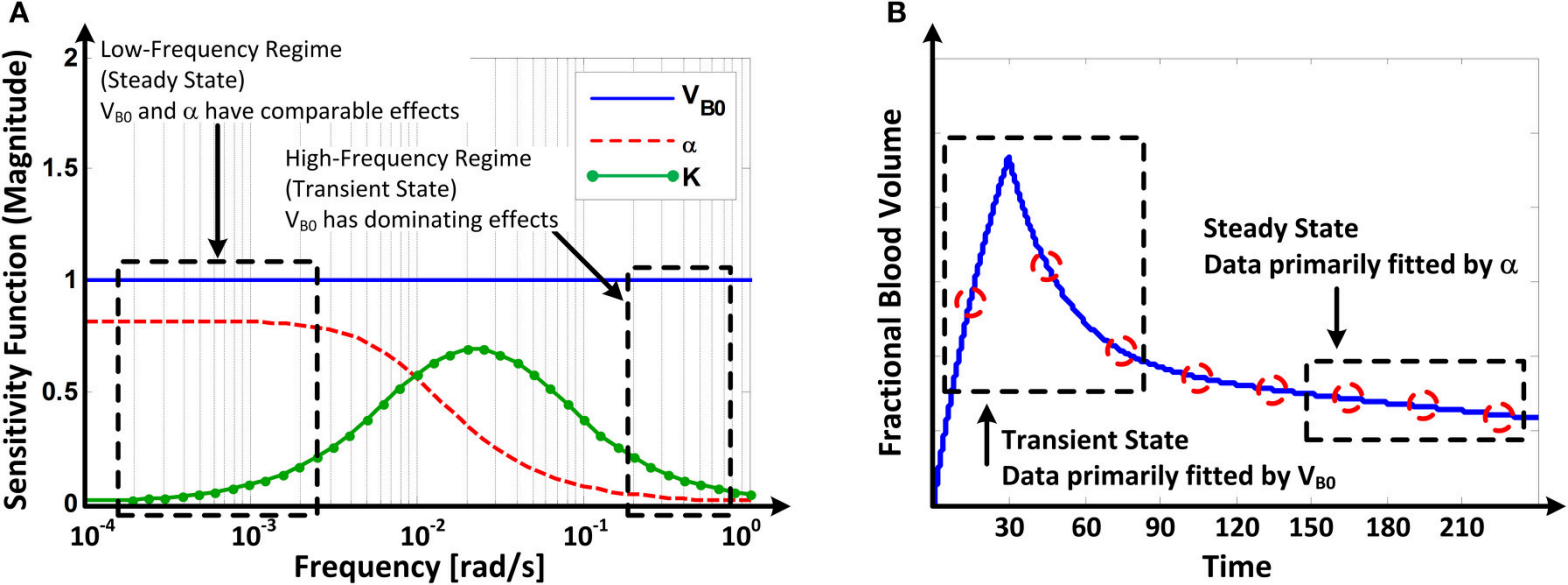
**(A)** Frequency responses of the model's sensitivity functions (Equation 12) to V_B0_ and α. **(B)** Mechanism of how V_B0_ and α are tuned in fitting the model to fractional blood volume response data.

Figure 6 illustrates **(A)** the frequency responses of the model's sensitivity functions *(12)* to V_B0_, K, and α and **(B)** the mechanism of how V_B0_, K, and α are tuned to fit the model to the data on ${\overset{⌣}{\text{V}}}_{\text{B}}$ response to u and v.

## Discussion

This paper presented a lumped-parameter model that can reproduce V_B_ response to u and v. This section considers the strengths and limitations of the model in terms of its parsimony, accuracy and physiological transparency.

### Lumped-parameter model

#### Parsimony

The lumped-parameter model presented in this paper is essentially a minimal model (Equation 5) characterized by only three parameters: α, K, and V_B0_. That this simple model can be fully characterized just by these parameters and accurately reproduce subject-specific V_B_ responses is its key benefit compared with the existing physiology-based first principles models (Pirkle and Gann, 1975; Mazzoni et al., 1988; Arturson et al., 1989; Carlson et al., 1996; Gyenge et al., 2003) whose complexity makes it extremely challenging to reproduce subject-specific V_B_ responses to u and v without exhaustive and impractical measurements.

#### Accuracy

The results of data analysis (Table 2 and Figures 3–5) support the ability of the lumped-parameter model to accurately reproduce V_B_ responses to u and v. The goodness of fit between measured versus model-reproduced ${\overset{⌣}{\text{V}}}_{\text{B}}$ responses is encouraging. These results, derived from five different fluids one of which was also associated with three different pre-infusion hemorrhage states (Dataset 1), suggest that the model may be suitable for summarizing V_B_ responses to a wide range of fluids over diverse V_B_ states.

#### Transparency

The proposed lumped-parameter model is a simple and highly structured model, constrained by the physiological principle that the ratio between the volume changes in the intravascular and interstitial fluid is regulated at a target value (Guyton et al., 1975). Thus, it is not obvious if the parameters estimated from the model fitting (Equation 9) would retain the intended physiological meanings. Since the actual values of the model parameters (V_B0_, K, and α) were not directly measured, the physiological transparency of the estimated parameters could not be established in a strict sense (i.e., by comparing the actual versus estimated parameters). Yet, the physiological “plausibility” of the proposed model is supported by a few observations from the data analysis (Table 2), as detailed below.

First, the values of ${\text{V}}_{\text{B}0}^{*}$ and α^*^ estimated by fitting the lumped-parameter model to measured ${\overset{⌣}{\text{V}}}_{\text{B}}$ responses were comparable, at least in terms of the orders of magnitude, to the known nominal plasma volume (~3.2 l for an 70 kg male; Skillman et al., 1967; Gyenge et al., 1999; Hedin and Hahn, 2005) and the ratio between the volume changes in the intravascular and interstitial fluid due to a perturbation in the body fluid volume (~2.3; Guyton et al., 1975; Lewis, 1986; Wears and Winton, 1990; Simpson et al., 1996): the values of α^*^ derived for the models associated with isotonic crystalloids (Datasets 1–2) ranged between 2.4 and 4.5, while the values of ${\text{V}}_{\text{B}0}^{*}$ derived for the models (Datasets 1–3) ranged between 3.1 l and 5.6 l.

Second, α^*^ decreased with an increase in the pre-infusion hemorrhage volume (Dataset 1), which is consistent with the physiological principle that more fluid is retained in the intravascular compartment as its volume decreases (Guyton et al., 1975). In fact, comparing the lower panels in Figure 3, the model estimated that q decreases as pre-infusion hemorrhage increases via a decrease in α^*^. At the same time, ${\text{V}}_{\text{B}0}^{*}$ also showed physiologically plausible behavior, exhibiting a decreasing trend with an increase in the pre-infusion hemorrhage (Dataset 1), meeting the physiological expectation that pre-infusion hemorrhage would decrease V_B0_.

Third, α^*^ estimated for colloids (Dataset 3) was smaller than α^*^ estimated for crystalloids (Datasets 1–2), which is consistent with the fact that colloid is better retained in blood than crystalloid due to its relatively large molecule size (Hahn, 2013) (note that this property of colloid should decrease α^*^ since less fluid is shifted to the interstitial compartment). In fact, comparing the lower panels in Figures 3–5, the model estimated that the fluid volume shifted from the intravascular to interstitial compartment relative to the change in V_B_ is largely smaller for colloids than crystalloids (note that the colloid infusion dose is only 40% of the crystalloid infusion dose; Table 1).

In sum, the lumped-parameter model presented in this paper is (1) simple in that it can be characterized by just three parameters; (2) able to reproduce V_B_ responses to u and v; and (3) showed physiologically plausible behaviors in terms of the estimated model parameters. We speculate that the lumped-parameter model may have clinical uses. For example, each time it is fitted to the response of a subject to bolus infusion of fluid, it may (1) characterize V_B_ state of a subject (e.g., by way of ${\text{V}}_{\text{B}0}^{*}$); (2) estimate unmeasured variables of interest, such as the volume of inter-compartmental fluid shift and the change in interstitial fluid volume; and (3) predict future physiological responses of a subject, such as predicting V_B_ response to future u and v. These speculative applications will be evaluated in future work.

### Parametric sensitivity analysis: mechanism underlying model fitting

Parametric sensitivity analysis provided in-depth insight as to how the model parameters are tuned to fit the model to the data. The Bode magnitude plots of the sensitivity functions (Equation 12) to V_B0_, K, and α suggest that the model's response is primarily affected by V_B0_ in the high frequency regime while comparably affected by V_B0_ and α in the low frequency regime, with the effect of K limited to mid-frequency regime (Figure 6A). Physically, this means that the model's transient response is largely affected by V_B0_ (and, to a modest extent, by K) while its steady-state response is affected comparably by both V_B0_ and α. Thus, in solving (Equation 9), the model tends to fit the transient portion of the response by primarily adjusting V_B0_, while it tends to fit the steady-state portion of the response by primarily adjusting α once a value of V_B0_ that best fits the transient response is determined (Figure 6B).

The above mechanistic insight on model fitting may elucidate why V_B0_ was often estimated to be larger than what is regarded as nominal as detailed below (see, e.g., V_B0_ estimated for Dataset 2 in Table 2). In response to a fluid bolus, V_B_ first peaks and then decreases as the fluid is distributed to the extravascular compartment. Thus, the real α may first peak and then decrease to a steady-state value as well (Guyton et al., 1975). However, the model assumes that the value of α is fixed at its steady-state (i.e., the lowest) value. The model (Equation 5) dictates that this α, together with actual V_B0_, yields an increase in the model-reproduced ${\overset{⌣}{\text{V}}}_{\text{B}}$ response. To compensate for this discrepancy, V_B0_ is tuned to be larger than its actual value to decrease the transient ${\overset{⌣}{\text{V}}}_{\text{B}}$ response. This may be (at least) a mechanism underlying the large values of V_B0_ derived for the Datasets 1–2. Compared with crystalloid (Datasets 1–2), V_B0_ derived for colloid (Dataset 3) were physiologically more plausible, because the infusion dose of colloid was small (Table 1), resulting in less likelihood of volume overload, thereby making the assumption of constant α more realistic.

### Study limitations

Despite promising initial results obtained for the proposed lumped-parameter model, there are a few limitations that must be investigated in more depth in future studies. First, the amount of data examined in this study was limited (seven datasets representing average subjects). Considering that the model is ultimately intended for use in individual subjects, the model must be assessed using datasets associated with individual (rather than average) subjects receiving fluid infusion under diverse physiological states. Noting that we only evaluated the model's ability to reproduce V_B_ response to fluid infusion (i.e., testing the model against the same data used for fitting), particular focus must be given to evaluate the model's ability to predict V_B_ response (i.e., testing the model against fresh data not used for fitting). Second, the physiological relevance of the model parameters was not fully established. Future work is required to compare estimated model parameters with their gold standard counterparts to assess the agreement between them. V_B0_ may be compared with its direct measurement, e.g., based on the spectrophotometric detection of indocyanine green (Henschen et al., 1993). α may be compared with the ratio between ΔV_B_ and ΔV_ISF_ in the steady state, which may be derived from the measurements of V_B0_ and ${\overset{⌣}{\text{V}}}_{\text{B}}$ as well as u and v. It is noted that α varies with volume state and is also altered by many physiological factors (Guyton et al., 1975). Thus, α must be interpreted as a resultant (and perhaps) linearized ratio between ΔV_B_ and ΔV_ISF_ within the physiological regime presented by the data, especially under the presence of diverse physiological changes. Third, blood hemoglobin concentration measurement can be subject to inaccuracy under large volume changes (Dasselaar et al., 2012). Future work must investigate the influence of the measurement inaccuracy on the quality of the estimated model parameters. Finally, the amount and attributes (e.g., frequency contents) of data required to accurately determine subject-specific model are yet to be examined. In this study, we have shown via the identifiability analysis that the model can be uniquely determined if the dataset is informative, and that model parameters estimated from dataset containing both transient and steady state exhibit physiologically plausible behaviors. Yet, for the model to be clinically useful, model parameters must be reliably determined from minimal data. Future analysis on this aspect will thus be necessary.

## Author contributions

RB, AR, and JH contributed to the development and validation of the lumped-parameter model as well as the manuscript preparation.

## Funding

This material is based on work supported by the Office of Naval Research (ONR) under Grant No. N000141410591 and N000141512018. Any opinions, findings, and conclusions or recommendations expressed in this material are those of the authors and do not necessarily reflect the views of the ONR.

### Conflict of interest statement

The authors have a pending patent related to the lumped-parameter model developed in this study.

## Appendix

### Volume kinetics model

For illustration purposes, a two-compartment volume kinetic model is considered. However, the finding applies to general volume kinetic models. The governing equation is given by (Hahn, 2010):

(A1) $$\begin{array}{r} {\overset{\cdot }{\text{v}}}_{\text{c}}={\text{R}}_{0}\;-\text{ C}{\text{L}}_{0}\;-\text{ CL}\frac{{\text{v}}_{\text{c}}\;-\;{\text{v}}_{\text{c}0}}{{\text{v}}_{\text{c}0}}\\ -\text{ C}{\text{L}}_{\text{d}}\left(\frac{{\text{v}}_{\text{c}}\;-\;{\text{v}}_{\text{c}0}}{{\text{v}}_{\text{c}0}}\;-\;\frac{{\text{v}}_{\text{t}}\;-\;{\text{v}}_{\text{t}0}}{{\text{v}}_{\text{t}0}}\right)\\ {\overset{\cdot }{\text{v}}}_{\text{t}}=\text{ C}{\text{L}}_{\text{d}}\left(\frac{{\text{v}}_{\text{c}}\;-\;{\text{v}}_{\text{c}0}}{{\text{v}}_{\text{c}0}}\;-\;\frac{{\text{v}}_{\text{t}}\;-\;{\text{v}}_{\text{t}0}}{{\text{v}}_{\text{t}0}}\right) \end{array}$$

where v_c_ and v_c0_ are the blood volume and its baseline value, v_t_ and v_t0_ the interstitial fluid volume and its baseline value, R_0_ fluid infusion rate, CL_0_, CL and CL_d_ the clearances associated with the baseline fluid loss, dilution-dependent elimination and inter-compartmental distribution, respectively. Denoting the changes in the intravascular and interstitial fluid volumes as state variables x_1_ and x_2_, (*Eq. A1*) is reformulated into the following:

(A2) $$\begin{array}{r} \left[\begin{array}{c} {\overset{\cdot }{\text{x}}}_{1}\\ {\overset{\cdot }{\text{x}}}_{2} \end{array}\right]=\left[\begin{array}{c} -\left({\text{K}}_{1}+{\text{K}}_{2}\right){\text{K}}_{3}\\ {\text{K}}_{2}-{\text{K}}_{3} \end{array}\;\right]\left[\begin{array}{c} {\text{x}}_{1}\\ {\text{x}}_{2} \end{array}\right]+\left[\begin{array}{c} 1\\ 0 \end{array}\right]\text{u} \end{array}$$

where K_1_ = CL/v_c0_, K_2_ = CL_d_/v_c0_, K_3_ = CL_d_/v_t0_ and u = R_0_ − CL_0_. So, taking the Laplace transform and solving for x_1_ and x_2_ yields:

(A3) $$\begin{array}{r} {\text{x}}_{1}\left(\text{s}\right)=\frac{\text{s }+\;{\text{K}}_{3}}{{\text{s}}^{2}\;+\;\left({\text{K}}_{1}\;+\;{\text{K}}_{2}\;+\;{\text{K}}_{3}\right)\text{s }+\;{\text{K}}_{1}{\text{K}}_{3}}\text{ u}\left(\text{s}\right),\\ {\text{x}}_{2}\left(\text{s}\right)=\frac{{\text{K}}_{2}}{{\text{s}}^{2}\;+\left({\text{K}}_{1}\;+\;{\text{K}}_{2}\;+\;{\text{K}}_{3}\right)\text{s }+\;{\text{K}}_{1}{\text{K}}_{3}}\text{u}\left(\text{s}\right) \end{array}$$

This results in the following:

(A4) $$\begin{array}{r} \frac{{\text{x}}_{2}\left(\text{s}\right)}{{\text{x}}_{1}\left(\text{s}\right)}\;=\;\frac{{\text{K}}_{2}}{\text{s }+\;{\text{K}}_{3}} \end{array}$$

Thus, the steady-state ratio between x_1_ and x_2_ (equivalent to α in our lumped-parameter model) reduces to lim_s → 0_x_2_(s)/x_1_(s) = K_2_/K_3_ = v_t0_/v_c0_, meaning that the ratio between the changes in the intravascular and interstitial fluid volumes is determined by the ratio of their initial values.
